# Rectal artesunate suppositories for the pre-referral treatment of suspected severe malaria

**DOI:** 10.1371/journal.pmed.1004312

**Published:** 2023-11-09

**Authors:** James A. Watson, Thomas J. Peto, Nicholas J. White

**Affiliations:** 1 Oxford University Clinical Research Unit, Hospital for Tropical Diseases, Ho Chi Minh City, Vietnam; 2 Centre for Tropical Medicine and Global Health, Nuffield Department of Medicine, University of Oxford, New Richards Building, Old Road Campus, Roosevelt Drive, Oxford, United Kingdom; 3 Mahidol Oxford Tropical Medicine Research Unit, Faculty of Tropical Medicine, Mahidol University, Bangkok, Thailand

## Abstract

In this Policy Forum article, James A. Watson and colleagues discuss recent guidelines relating to pre-referral treatment of suspected severe malaria with rectal artesunate suppositories in remote areas.

**Summary points**

- The World Health Organization Malaria Policy Advisory Group (WHO MPAG) has recently advised against deployment of rectal artesunate suppositories (RAS) for the treatment of severe malaria in remote areas where efficient referral to hospital is not possible.
- Untreated severe malaria is almost always fatal. In these remote areas, no RAS availability will very likely mean no treatment at all. These are the areas where childhood mortality from malaria is greatest.
- The earlier artesunate is given in the course of severe malaria illness, the greater the life-saving benefit.
- Most of the life-saving benefit of artesunate follows the first dose. The route of administration does not change the antimalarial effect of artesunate.
- In places where referral is not possible, treatment with RAS only, followed by oral artemisinin combination therapy when the patient can take oral medications, is likely to be sufficient in most cases.
- Selection of artemisinin resistance following a single RAS dose is highly unlikely.
- Bacterial septicaemia is frequently misdiagnosed as severe malaria. There are no broad-spectrum antibiotics that can be rectally administered—a major therapeutics gap for community treatment of severe febrile illness.
- We urge the WHO MPAG to reconsider their recommendation against deployment of RAS in places where efficient referral to hospital is not possible and to promote development of the community health worker infrastructure that would support effective deployment of RAS in remote areas where it will save the most young lives.

## Introduction

**Fig 1:**
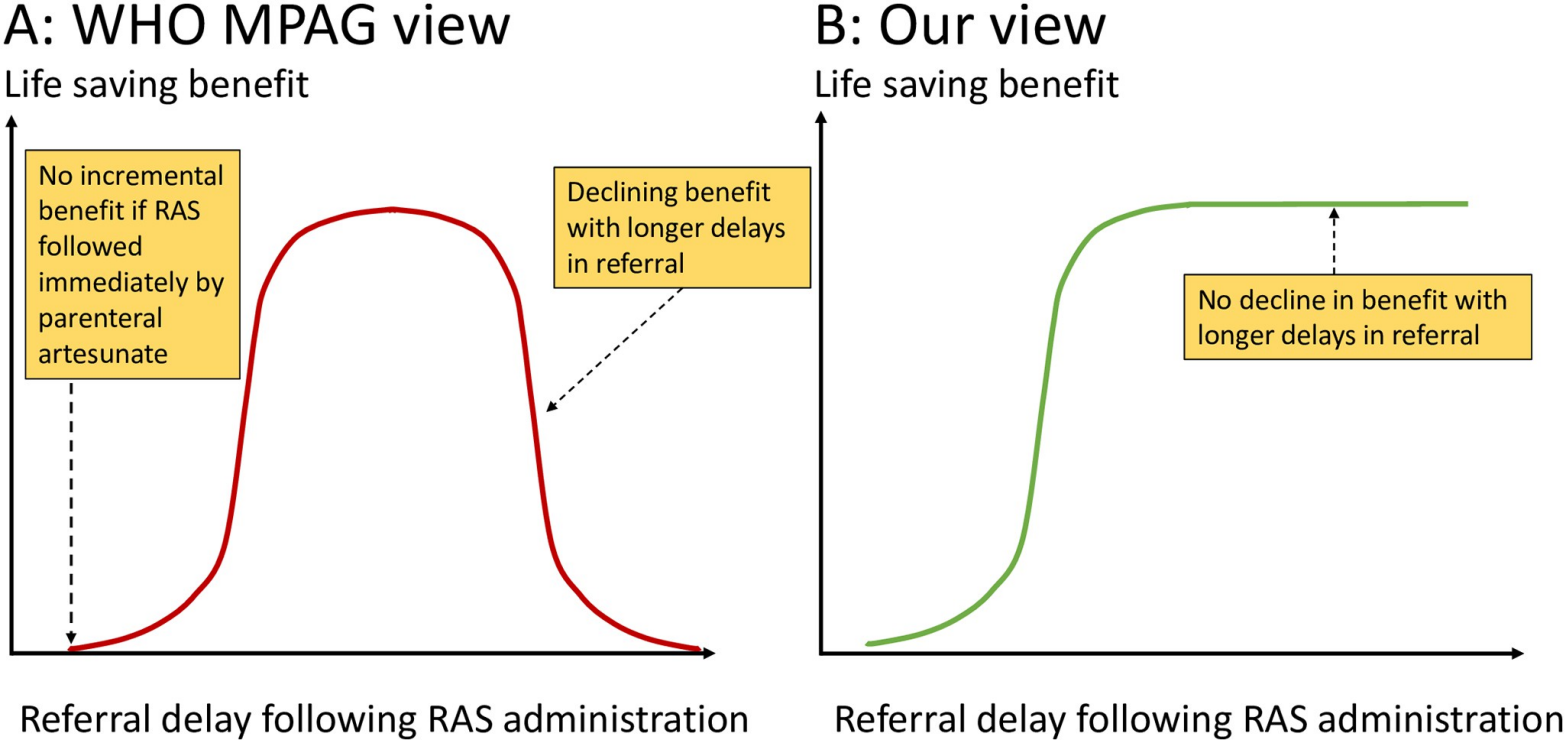
The 2 different views on the life-saving benefits of giving pre-referral rectal artesunate (RAS) to a child with severe malaria. Note that there is little or no additional benefit if RAS is followed immediately by parenteral artesunate. In the view of the WHO MPAG (A), there is also no benefit if referral is delayed. This results in a parabolic relationship between delay in referral and life-saving benefit. In our view (B), the benefits do not decline with delay (until either death or spontaneous recovery) so the relationship is sigmoid.

Malaria is a major cause of preventable death in childhood in tropical countries. This is one of the main justifications for the substantial global investment in malaria control. There are currently 2 fundamentally different views on how to reduce the mortality of children with severe malaria in remote or inaccessible areas where most deaths occur. The first view is that severe malaria mortality in remote areas can be reduced only by providing an improved continuum of care from the community to the hospital. The other view is that the greatest immediate impact on mortality will come from prompt administration of a highly effective antimalarial to seriously ill children in or near their homes (Fig 1). In the former view, providing rectal artesunate suppositories (RAS) in the community will not save lives unless it is followed by prompt referral to a hospital or health centre where follow-up parenteral artesunate treatment is provided. In the latter view, most of the life-saving benefit is provided by the single community administered rectal artesunate dose. If this latter view is correct, then the benefit will be greatest in the hardest to reach places where the death toll is highest [1]. Although referral is clearly desirable for patients who are unable to tolerate oral medications or are severely ill, many patients will soon be able to tolerate oral medication after receiving RAS. Thus, if referral is not practicable, or the transport itself incurs significant risk, then follow-up oral treatment can be given in the community. Referral to hospital is not therefore a prerequisite for administration of RAS.

These 2 divergent views have very different policy implications. It appears that the World Health Organization Malaria Policy Advisory Group (WHO MPAG) supports the first view. WHO MPAG emphasises “the critical importance of countries focusing on readiness to provide an effective continuum of care as a ***prerequisite*** for introduction of RAS” [2]. WHO MPAG advises restricting RAS deployment only to areas with good patient referral systems, stating that:

*To reduce the CFR [case fatality ratio] for children with severe malaria*, *there needs to be a functional continuum of care for severely ill children*, *with a good referral system and referral facilities equipped to comprehensively manage a severely sick child* [3].

We argue that this view is incorrect and will result in preventable childhood deaths.

This advice follows a critical review commissioned by WHO MPAG of the available evidence on RAS [3], notably of the recent Community Access to Rectal Artesunate for Malaria (CARAMAL) study—a large observational study of RAS deployment conducted in Nigeria, Democratic Republic of Congo, and Uganda. CARAMAL reported that RAS deployment was associated with a 4-fold increase in childhood mortality in Nigeria. In response to the preliminary release of these findings in January 2022, without further consultation, WHO advised a moratorium on RAS deployment [4]. Since then, the weaknesses and likely biases in the CARAMAL study have been discussed at length [3,5–8]. The independent review commissioned by WHO concluded that a causal relationship between RAS deployment and increased mortality in the CARAMAL study was not supported by the study design and data collection [3]. Following this report, in April 2023, the MPAG repositioned its stance and now concludes that the results of the CARAMAL observational study emphasise that provision of an effective continuum of care is a necessary ***prerequisite*** for the introduction of RAS [2,3]. A field manual is now under preparation “which clearly outlines the conditions under which the introduction of RAS can be effective” [2]. In effect, the MPAG advised moratorium on RAS has not been lifted. WHO MPAG indicates that RAS can only be effective if deployed in settings with a seamless and efficient referral system, and that if such a referral system is not in place (which today applies to the majority of remote malaria endemic areas), then RAS should not be deployed. We think this is fundamentally wrong [1]. The greatest benefit from RAS in terms of the number of childrens’ lives saved is likely to be in settings where the health systems are weakest. That is where most malaria deaths occur.

## The development of rectal artesunate

Artemisinin suppositories were first used to treat falciparum malaria in China in the 1970s [9,10]. Studies in China, and then in Viet Nam, showed that the rectal route of administration was highly effective in the treatment of severe malaria [9–14]. These positive results led to the development by WHO Tropical Disease Research programme (TDR) of rectal artesunate—a rectally administered gel formulation in a soft capsule. Rectal formulations provide an easy route of drug administration which, in remote areas, can be given by community health workers to patients who are unable to take oral medications (and in situations where parenteral administration is not possible). By 2010, parenteral artesunate had become the treatment of choice for severe falciparum malaria throughout the world [15] following definitive results from large randomised clinical trials [16–18]. Concurrently, a large, randomised, placebo-controlled trial of RAS administered as pre-referral treatment of suspected severe malaria by community health workers was carried out in Africa and Asia (Study 13) [19]. Severe malaria cannot be distinguished clinically from other life-threatening infections so many enrolled patients did not have malaria. The overall results of study 13 were not conclusive. A clear mortality benefit (an approximate 25% reduction) was shown only in children who received RAS and were parasitaemic (i.e., who really did have severe malaria) and who took more than 6 h to reach hospital, a post hoc subgroup analysis. In the small subgroup of adults studied in Bangladesh, there was higher mortality in the group treated with RAS compared to placebo (22 versus 9 deaths). This is considered most likely a chance finding. But since then RAS have not been recommended in adults, and an upper age limit of 6 years has been imposed, although there is no known reason why RAS would not be effective in adults. Over the past decade, no evidence of serious artesunate toxicity has emerged in millions of drug administrations. The deployment of parenteral artesunate has been credited with saving hundreds of thousands of lives but unfortunately, the development and deployment of RAS has been very slow. WHO prequalification of quality-assured RAS occurred only in 2018 (3 decades after it was first suggested for use in primary health care settings). Then, in January 2022, WHO MPAG recommended abruptly that the RAS roll out be halted [4].

## Operational feasibility

**Fig 2:**
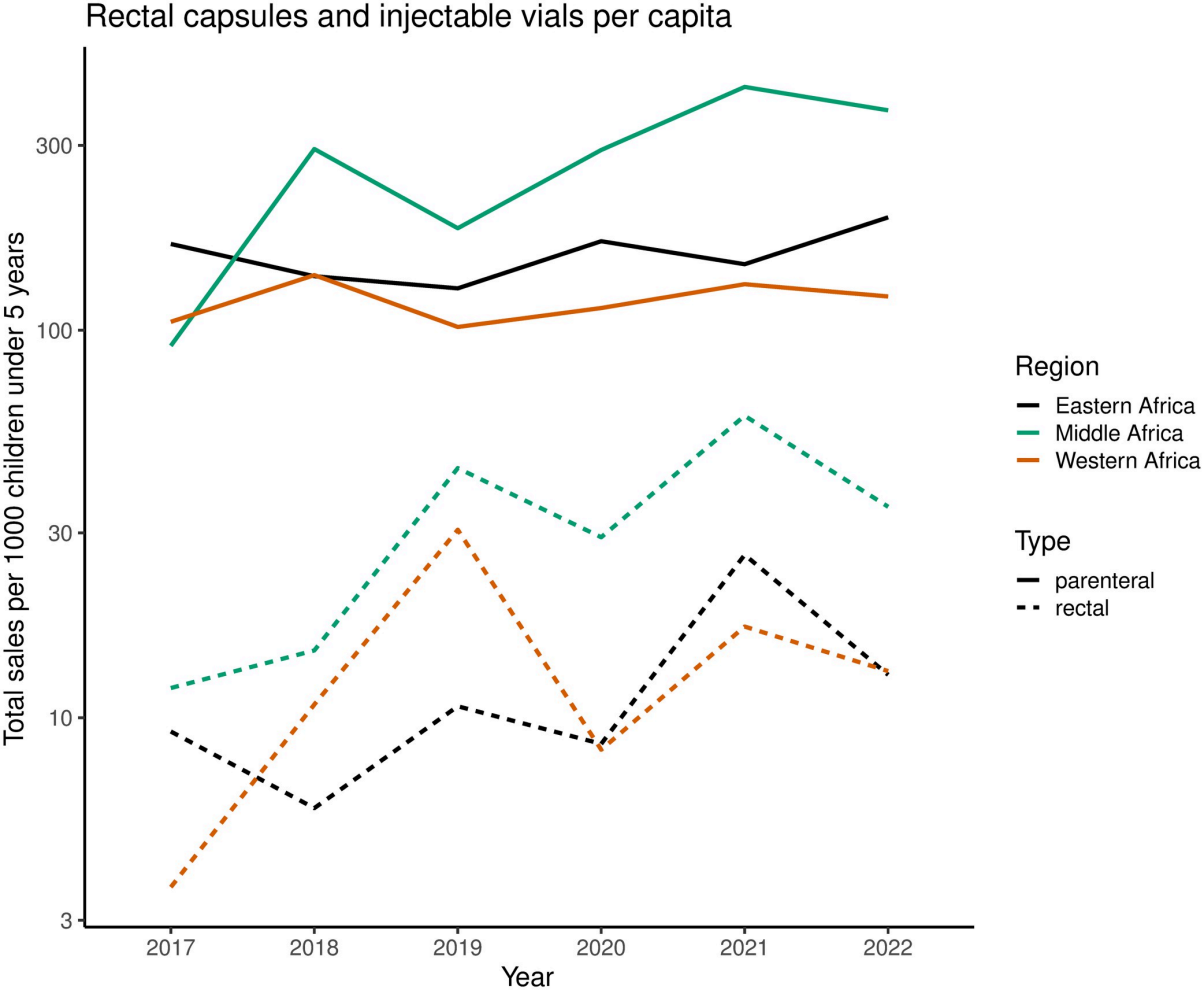
Sales of rectal artesunate capsules (50 and 100 mg base doses combined) and injectable artesunate vials (30, 60, and 120 mg base doses combined) per capita for children under 5 years of age. Data are aggregated by United Nations Department of Economic and Social Affairs subregions.

The most widely used RAS formulation is a rectal capsule consisting of a soft capsule shell (gelatin, glycerol, and titanium dioxide) and the fill blend (hard fat, medium chain triglyceride that contains 100 mg artesunate). These rectal capsules should not be stored above 25°C, in particular above 30°C. The relatively short shelf-life (2 years) and storage temperature requirement is a limitation for many malaria endemic areas where ambient temperatures commonly exceed 30°C. The dose for ages 6 months to <3 years is 1 capsule and for children 3 to 6 years is 2 capsules. The rectal capsules are simple to administer and well tolerated. In sub-Saharan Africa, since 2017, an estimated 1.4 million rectal artesunate capsules per year were sold by the 2 major WHO-prequalified manufacturers (Strides Shasun and Cipla). Using UN population estimates for the number of children under 5 by country, this corresponds to approximately 10 to 30 capsules per 1,000 children under 5 years of age (Fig 2). In comparison, a total of 25 million injectable artesunate vials per year were sold by the same manufacturers (between 100 to 300 vials per 1,000 children under 5 years, an underestimate of the total sales as this does not include Fosun Pharma).

## Pharmacology of RAS

The main pharmacokinetic disadvantage of RAS is its variable absorption (>1,000 fold variation in peak dihydroartemisinin concentrations) [20]. The overall approximate 25% rectal bioavailability is offset by the 3- to 4-fold higher dose [21]. Absorption is rapid (average time to peak concentrations approximately 30 min). Artesunate is hydrolysed rapidly in plasma to its biologically active metabolite dihydroartemisinin (DHA), which is then eliminated rapidly with a half-life of less than 1 h. DHA accounts for the majority of the antimalarial effect. There is no evidence that the route of drug administration affects the pharmacodynamic properties of artesunate. Once they are in the blood, both artesunate and DHA have the same parasiticidal properties irrespective of the administration route. These drugs are extremely well tolerated. Their main pharmacodynamic advantage is their broad stage specificity of antimalarial action [22,23]. This results in rapid reduction in parasitaemia and, critically for their life-saving action, prevention of further parasitised erythrocyte cytoadherence. Pharmacometric evaluation suggests that near maximal effects are obtained in the majority of treated patients [20]. In clinical trials, the greatest therapeutic benefit compared with quinine is seen in patients with large numbers of circulating parasites [17,24]. Preventing these circulating parasites from sequestering reduces the risk of potentially lethal microvascular obstruction and dysfunction, and thus the risk of death [25,26]. The ring stages of *P*. *falciparum* circulate for 12 to 18 h, so a 12-h delay in giving artesunate to a patient with severe malaria allows nearly all the parasites that were circulating at the time of diagnosis to have matured and sequestered. This is why delays in providing artesunate treatment are so dangerous. RAS were developed specifically because such delays are very common in remote areas of the tropics where most malaria deaths occur. Delay in parenteral administration is therefore critical to the life-saving benefit of RAS. The converse is that RAS provides little additional benefit if parenteral treatment is given soon afterwards.

## The life-saving benefit of RAS derives mainly from the first dose

Once falciparum malaria has progressed to cause vital organ dysfunction, most patients will die if untreated. Progression of severe disease is rapid, particularly in children, so the window of opportunity to save life is relatively narrow [26]. If untreated severe malaria is fatal, then it follows logically that the longer the expected delay in hospitalisation and receipt of parenteral antimalarial drugs, the greater the benefit from pre-referral treatment. Thus, RAS are likely to be most beneficial when referral is delayed or impossible: where the alternative is no treatment. It is important to emphasise this point—in many rural areas where referral is difficult or impossible, no RAS will mean no treatment at all.

Patients with severe malaria usually have a high parasite biomass (>10^10^ parasites/kg). Therapeutic concentrations of artesunate and the active metabolite dihydroartemisinin following a single dose (irrespective of administration route) result in a 10,000-fold reduction in parasite biomass within 1 asexual parasite cycle (circa 48 h) [22]. Even within 24 h circulating parasite numbers have been reduced by approximately 50-fold. The relatively tiny 48 h post-treatment in vivo parasite biomass of between 10^7^ and 10^8^ parasites is equivalent to that associated with the pyrogenic density in a non-immune host (i.e., it is on the threshold of the biomass that causes fever in malaria). It is lower than densities usually found in patients with uncomplicated malaria in endemic areas. If it were not preceded by a much higher biomass, this biomass would not cause symptoms in many people. In the hospital, it is recommended that a second dose of parenteral artesunate is given at 12 and 24 h after the first to ensure that maximal antimalarial effects are obtained. This can be considered as a therapeutic “insurance policy.” But laboratory studies and clinical studies both suggest that near maximal antimalarial effects are obtained with a single dose and thus single exposure [27–31].

What would happen if, following the single RAS administration, no further antimalarial treatment was given? Assuming a highly efficient and unrestricted parasite multiplication rate of 10-fold per cycle, it would then still take another 8 days for the patient to regain a life-threatening parasite biomass similar to that which preceded the first dose (i.e., 10 days in total) [32]. Thus, even in a worst case scenario, the single rectal dose has bought at least 1 week’s worth of protection. If severe malaria is the true cause of severe illness, the patient’s condition should improve allowing for oral treatment with a full course of an artemisinin-based combination therapy (ACT). So while the single RAS dose is not curative, the pharmacological evidence strongly suggests that nearly all the life-saving benefit in severe malaria results from this first dose. The policy implication of these pharmacological observations is that administration of rectal artesunate should have a substantial impact on the mortality of severe malaria in the most hard to reach places where the death toll is highest, whether or not it is followed by definitive treatment. It is important to emphasise that this is not a recommendation against referral to hospital, which is to be strongly encouraged if it is safe and feasible. Good intensive care can save lives in severe malaria. Furthermore, a single dose of artesunate is not curative, and recrudescence is inevitable. RAS must be followed by a full course of an ACT. Our point is simply that most of the substantial life-saving benefit from RAS is not dependent on successful referral and subsequent management.

## Most severely ill African children do not have severe malaria

One important practical point is that severe falciparum malaria is substantially overdiagnosed in African children [33]. Even in experienced research centres, it is estimated that about one third of children diagnosed originally as having severe malaria actually have another cause of their life-threatening illness (most likely sepsis) [34]. In ordinary health centres and hospitals, this proportion is presumably even higher. Bacterial septicaemia is common and carries a higher case-specific mortality than severe falciparum malaria. RAS does not benefit a child with bacterial septicaemia. There are currently no broad-spectrum antibiotics that can be rectally administered, a major issue for community treatment of severe febrile illness. Thus, when referral is possible, it is clearly preferable.

## How important is the resistance risk?

Incomplete antimalarial treatment can engender resistance [35]. This is because it provides a selective advantage to resistant parasites, which then have a greater chance of causing a recrudescent infection and then transmitting to other people. There are 2 stages of this process. First, the rare emergence of de novo resistance, and second, the enhanced spread of established resistance. Most de novo-resistant parasites are not transmitted because they comprise a tiny proportion of the infecting biomass and do not generate transmissible densities of gametocytes carrying the resistance genes [35]. Resistance only “counts” if it is transmitted. A single artesunate dose provides very little transmission advantage because, even if a highly resistant mutant is selected, it cannot reach competitive numbers within 1 asexual life-cycle. Spread of resistance is also very unlikely as a single dose is not curative even in sensitive infections, so there is very little selective advantage. Repeated single dose administrations with inter-dose re-expansion of the parasite biomass is necessary to allow a selected resistant mutant to reach sufficient densities that will allow onward transmission [32]. In summary, a single dose of artesunate provides very little resistance selective pressure. It should be noted also that the number of cases receiving pre-referral treatment of severe malaria is very small in comparison with the much larger number of febrile illnesses receiving oral antimalarial treatment.

## Deployment

Ideally, RAS should be administered by a village health worker who lives close by in order to minimise delays in the treatment of suspected severe malaria. In remote, hard to reach areas of Asia, supporting village health workers to diagnose and treat malaria has been very effective in reducing the burden of malaria [36]. Unfortunately, village health worker networks are not well developed in rural Africa. Instituting or strengthening such networks would have a substantial benefit for the treatment of malaria and other acute infectious diseases. These same health workers could also be trained to use malaria rapid diagnostic tests and supplied with oral antimalarial ACTs (both for the management of uncomplicated malaria, and also so that consolidation treatment is available locally if a patient cannot be referred). Health service strengthening is clearly a major task of great importance, but one that should accompany rather than precede RAS deployment.

## Conclusions

Untreated severe malaria is nearly always fatal. Providing RAS in the remote, difficult to reach, or poorly served locations where childhood malaria mortality is highest will save many more lives than restricting it to those well-served places with “a good referral system and referral facilities equipped to comprehensively manage a severely sick child” [3]. We consider that the WHO MPAG position on RAS deployment is not supported by evidence. It will prevent the children who need RAS the most from receiving it. Of course, referral systems and the health sector in malaria endemic areas should be strengthened. This has been a public health objective for decades. But to wait for this before deploying RAS makes no sense. The perfect is the enemy of good. RAS was developed specifically because of these health system weaknesses. Severe malaria requires urgent administration of artesunate. Further delays in RAS deployment will result in more preventable deaths in African children. We strongly believe the WHO MPAG advised restrictions on the deployment of RAS are harmful and should be lifted immediately.

## Abbreviations

ACT: artemisinin-based combination therapy
CARAMAL: Community Access to Rectal Artesunate for Malaria
RAS: rectal artesunate suppositories
WHO MPAG: World Health Organization Malaria Policy Advisory Group
